# Supplementary material for: Prevalence and early-life determinants of mid-life multimorbidity: evidence from the 1970 British birth cohort
Source: BMC Public Health. 2021 Jul 28;21:1319. doi: 10.1186/s12889-021-11291-w (PMC8317357; doi:10.1186/s12889-021-11291-w)
Supplement: Supplementary file 1 — Additional file 1: Table S1. Description of self-reported outcome variables under multimorbidity definition. Table S2. Description of objectively measured outcome variables under multimorbidity definition. Table S3. Description of the exposures. Table S4. Description of potential confounding. Table S5. Key characteristics of main relevant studies. Supplemental text 1. Missing data strategy. Table S6. Frequency and predictors of missing data in the exposures and the outcome. Table S7. Multimorbidity prevalence at age 46–48 based on different sample definitions. Table S8. The risk ratio and E-values for the association between each exposure and multimorbidity at age 46–48 in the fully adjusted models. Table S9. Association between early-life exposures and multimorbidity at age 46–48 in the sample with complete cases only and using a multimorbidity definition without mental health morbidity. [file 12889_2021_11291_MOESM1_ESM.docx]

Supplemental table 1. Description of self-reported outcome variables under multimorbidity definition.

Supplemental table 2. Description of objectively measured outcome variables under multimorbidity definition.

Supplemental table 3. Description of the exposures.

Supplemental table 4. Description of potential confounding.

Supplemental table 5. Key characteristics of main relevant studies.

Supplemental text 1. Missing data strategy.

Supplemental table 6. Frequency and predictors of missing data in the exposures and the outcome.

Supplemental table 7. Multimorbidity prevalence at age 46-48 based on different sample definitions.

Supplemental table 8. The risk ratio and E-values for the association between each exposure and multimorbidity at age 46-48 in the most adjusted models.

Supplemental table 9. Association between early-life exposures and multimorbidity at age 46-48 in the sample with complete cases only and using a multimorbidity definition without mental health morbidity.

| Supplemental table 1. Description of self-reported outcome variables under multimorbidity definition. | | | |
| --- | --- | --- | --- |
| Variable | Type of variable | Age | Description |
| Self-reported chronic morbidity | Outcome | 46-48 | The questionnaire was self-administered with the computer-assisted personal interviewing (CAPI).  The question was: “Since the last interview/4 years ago have you had any of the health problems listed on this card? Please include any health problems that had already started before that date.”  - Asthma or wheezy bronchitis; convulsion, fit, epileptic seizure; recurrent backache, prolapsed disc, sciatica or other back problem; cancer or leukaemia; problems with hearing; problems with eyes (do not include problems which are resolved by wearing glasses or contact lenses – e.g., short-sightedness, long-sightedness or astigmatism); heart problems; chronic fatigue syndrome (ME); liver disease including viral hepatitis B or C; arthritis; stroke. |
| Drinking | Outcome | 46-48 | Drinking behaviours were measured with the Alcohol use disorders identification test for primary care (AUDIT PC), including five questions.^1^ A score of five or more indicates high-risk drinking.^2^ The test was found to have 98.3% sensitivity 90.9% specificity for detecting hazardous drinkers in randomly selected primary care patients.^2^ |
| Mental health morbidity | Outcome | 46-48 | BCS70 at age 46-48 used the Malaise Inventory,^3^ which was self-administered with the computer-assisted personal interviewing (CAPI). A shorter 9-item version (as opposed the full 24 items version) was used, with a binary (“yes-no”) response scale. It showed robust psychometric properties when tested in the general population: Cronbach’s alpha = 0.70 – 0.80; all items identify a common factor; AUC against self-reported diagnosed psychiatric morbidity = 0.77–0.79).^4^ |

| Supplemental table 2. Description of objectively measured outcome variables under multimorbidity definition. | | | |
| --- | --- | --- | --- |
| Variable | Type of variable | Age | Description |
| Hypertension | Outcome | 46-48 | Hypertension was defined as SBP≥140 mmHg or DBP≥90 mmHg or taking medications for high blood pressure. These medications included β-blockers, drugs affecting the renin-angiotensin system, calcium channel blockers and diuretics. Systolic and diastolic blood pressure was measured in a seated position, after 5 min rest, using automated oscillometric devices (NCDS: Omron HEM 705; BCS70: OMRON HEM 907). A large cuff was used for participants with a mid-upper arm circumference of 32 cm. The measurement was repeated three times and all successful and reliable measures were averaged to obtain final blood pressure values (BCS70: n=6,970).^5^ The measurement was taken on the left arm. Pregnant women were excluded. |
| Diabetes | Outcome | 46-48 | Diabetes was indicated as Glycated Haemoglobin (HbA1c) of 48 mmol/mol (6.5%) or over or taking medications for diabetes ^6^. Glycated haemoglobin is an integrated measure of the level of sugar in the blood over the previous eight to 12 weeks before measurement. HbA1c is regarded as a useful screening tool for detecting diabetes in the general population.^7^  HbA1C was measured using whole blood supplied in ethylenediaminetetraacetic acid (EDTA) tube and was analysed using the Tosoh G8 analyser. Exclusion criteria included: people with clotting or bleeding disorder; people who were currently on anticoagulant drugs, e.g., Warfarin therapy; people who have ever had a fit; people who were not willing to give their consent in writing; pregnant women; respondents who were HIV positive or who have hepatitis B or C. |

| Supplemental table 3. Description of the exposures. | | | |
| --- | --- | --- | --- |
| Variable | Type of variable | Age | Description |
| Birthweight | Exposure/confounding | 0 | Birthweight of each cohort member was measured in ounces and converted into kilograms. |
| Cognitive ability | Exposure/confounding | 10 | The cognitive ability was assessed by a modified version of the British Ability Scales^8^ comprising four sub-scales: word definitions and word similarities were used to measure verbal ability, and recall of digits and matrices was used to measure non-verbal ability. A principal component analysis (PCA) was conducted for each of the verbal and nonverbal subtests, in order to attain a general cognitive ability factor (g). Following the protocol of previous studies, we saved scores from the first unrotated factor for each valid case.^9^ We summed up the individual items to derive an overall score for each sub-test and we conducted a PCA on these four variables, again saving the first components score (accounting for 57% of total variance). The scores were standardised to a mean of 0 and a standard deviation of 1. |
| Body mass index (BMI) | Exposure/confounding | 10 | Height and weight were measured by trained medical personnel using standard protocols at age 10. The weight and height measures were harmonised by the CLOSER consortium to facilitate comparisons across cohorts.^10^ |

| Supplemental table 3 (cont.). Description of the exposures. | | | |
| --- | --- | --- | --- |
| Variable | Type of variable | Age | Description |
| Externalising problems | Exposure/confounding | 16 | The modified version of the Rutter A scale, a measure of mental health capturing conduct problems, hyperactivity, emotional and peer problems, was completed by mothers of the participants as part of the home interview.^3^  As in previous studies;^11^ two scales were created: 1) externalising problems (five items, e.g., “destroys own or others belongings”) and internalising problems (three items, e.g., worries about many things). Each item has a 3-point response scale (“Not true”=0, “Somewhat true”=1, “Certainly true”=2). Hence, a higher score reflects more externalising or internalising problems. The measure was tested in the general population; acceptable inter-rater reliability (r=0.64) and retest reliability (r=0.74).^3^ |
| Internalising problems | Exposure/confounding | 16 |  |
| Father’s social class at birth | Exposure/confounding | 0 | Occupation of the father at the time of the participants’ birth was coded according to the classification Socio-economic Groups (SEG) classification introduced in 1951. Current or most recent jobs of participants’ fathers were classified as: we (professional), II (managerial and technical), III (skilled non-manual/manual), IV (partly-skilled), and V (unskilled); with those classified as “missing” who had unclassifiable occupation/had insufficient information/served in armed forces/were unemployed or sick or retired. |

| Supplemental table 4. Description of potential confounding. | | | |
| --- | --- | --- | --- |
| Variable | Type of variable | Age | Description |
| Gender | Confounding/moderator | 0 | A binary variable (men vs women) – recorded at birth; if the information was missing it was completed with records from age 10 and 42. |
| Gestational age | Confounding | 0 | Gestational age measured in days since the start of the last menstrual period was recorded at birth and converted into completed weeks and used as a continuous variable. |
| Smoking during pregnancy | Confounding | 0 | All variables are self-reported by the mother at birth, except for the number of children in the household (at five years old). “Unmarried” category is only based on marital status and includes cohabitation. |
| Mothers’ height | Confounding | 0 |  |
| Mother’s unmarried at birth | Confounding | 0 |  |
| Parental divorce | Confounding | 0-16 | The variable was derived using the information on change of marital circumstances collected from mother between birth and age 16. In addition, it was validated with the question about the age of divorce asked to cohort members. |
| Mother’s mental health | Confounding | 10 | When the study members were age 10, their mothers were asked a series of questions about their own mental health using a 24-item Malaise inventory.^3^ The 24 items were scored from 0 to 100 with 0 reflecting seldom or never and 100 most of the time. The 24 items were added together to create a continuous scale from 1 to 2154, with a higher score reflecting higher levels of depression. |

| Supplemental table 4. (cont.) Description of potential confounding. | | | |
| --- | --- | --- | --- |
| Variable | Type of variable | Age | Description |
| Tenure* | Confounding | 5-10 | This variable indicates whether the study member was in rented or owned accommodation at age 5 and 10. It does not distinguish between accommodation rented privately and rented from the council. |
| Overcrowding* | Confounding | 5 | It is an indicator of whether the study member experienced overcrowding in childhood (>1 person per room), measured as the number of persons per room. The measure is a median score of overcrowding collected at different points at 5 years of age. |
| Teen mother* | Confounding | 0 | The variable is defined using the age of the mother when the study member was born. All those mothers under the age of 20 were identified as teen mother. |
| Breastfeeding* | Confounding | 5 | The variable includes details on whether the study member was breastfed, as reported by the mother. |
| Parental interest in child’s education* | Confounding | 10 | Parental interest in schooling was reported by the child’s teacher at age 10 (high interest vs moderate interest vs low interest). The interest of the parent who was most interested in their child’s education was used. If either parent had missing data, then the response of the parent which had data was used. |
| Length of time absent from school due to illness* | Confounding | 10 | Parents were asked how long the study member had been away from school due to ill health in the past 12 months. |
| * The variable were harmonised across cohorts as part of CLOSER work package 2.^12^ | | | |

| Supplemental table 5. Key characteristics of main relevant studies. | | | |
| --- | --- | --- | --- |
| **Reference** | **Country; Study (period)** | **Exposure (age)->Outcome (age): strength of the association (SE/CI95%)** | **Adjusted confounding (age)** |
| Belbasis et al. (2016)^13^ | Umbrella review of systematic reviews and meta-analyses (n=39) | - Highly suggestive evidence: Lower birthweight->All types of leukaemia, overweight or obese (16+) | Importance of gestational age emphasised. |
| Birnie et al. (2016)^14^ | Meta-analysis (n=19) | - Low childhood SEP (vs high)-Grip strength (18+):  -0.13 standard deviations (95% CI: -0.06, -0.21)  - Low childhood SEP (vs high)->Chair rise time (18+): 6% (4%, 8%) higher  - Low childhood SEP (vs high)->Inability to balance for 5s (18+): OR=1.26 (1.02, 1.55) | Age. |
| Booth et al. (2013)^15^ | Clinical Practice Research Datalink (2005-2011) | - Obese category I (30+)->Multimorbidity (30+): OR=2.04 (1.98 to 2.11) | Age; gender; socioeconomic deprivation; smoking. |
| Buchanan et al. (2002)^16^ | Great Britain; NCDS (born 1958) | Men:  - Internalising problems (7)->Psychological distress (33): OR=1.0 (0.57–1.90)  - Externalising problems (7)->Psychological distress (33): OR=1.9 (1.11–3.30)  Women:  - Internalising problems (7)->Psychological distress (33): OR=1.2 (0.86–1.81)  - Externalising problems (7)->Psychological distress (33): OR=1.7 (1.03–2.70) | Gender and parental socioeconomic status when the child was born; parental mental health (7); structure of the parental background; social disadvantage; experience of care (7); family involvement with the police/probation service (7); agency referral for difficulties at school (7); social services involvement and domestic tension (7); outings with mother (7); father reads to child (7); child’s good numeric and creative skills (7). |

| Supplemental table 5. (cont.) Key characteristics of main relevant studies. | | | |
| --- | --- | --- | --- |
| **Reference** | **Country; Study (period)** | **Exposure (age)->Outcome (age): strength of the association (SE/CI95%)** | **Adjusted confounding (age)** |
| Cooper & Power (2008)^17^ | Great Britain; NCDS (born 1958) | Men:  - Lower birthweight->Higher total cholesterol (44-45): B=0.01 (−0.02 to 0.05)  - Lower birthweight->Higher LDL-cholesterol (44-45): B=0.03 (−0.01 to 0.06)  - Lower birthweight->Higher HDL-cholesterol (44-45): B=0.02 (0.01 to 0.03)  - Lower Birthweight->Higher triglycerides (44-45): B=−0.04 (−0.06 to −0.02)  Women:  - Lower birthweight->Lower total cholesterol (44-45): B=−0.07 (−0.10 to −0.03)  - Lower birthweight->Lower LDL-cholesterol (44-45): B=−0.03 (−0.06 to −0.002)  - Lower birthweight->Higher HDL-cholesterol (44-45): B=0.01 (−0.005 to 0.02)  - Lower birthweight->Lower triglycerides (44-45): B=−0.05 (−0.07 to −0.03) | Gestational age; smoking status; alcohol use; physical activity levels; indicators of lifetime socioeconomic position; menopausal status; height, BMI. |
| Galobardes et al. (2006)^18^ | Systematic review (n=40) | - Childhood SEP->Higher risk of cardiovascular disease and coronary heart disease | Association tended to remain after adjusting for adult SEP. |

| Supplemental table 5. (cont.) Key characteristics of main relevant studies. | | | |
| --- | --- | --- | --- |
| **Reference** | **Country; Study (period)** | **Exposure (age)->Outcome (age): strength of the association (SE/CI95%)** | **Adjusted confounding (age)** |
| Hardy et al. (2003)^19^ | Great Britain; NSHD (born 1946) | - Birthweight (kg)->Systolic blood pressure (36): B=-1.86 mm Hg  (-2.90 to -0.82; p<0.01)  - Birthweight (kg)->Systolic blood pressure (43): B=-2.09 mm Hg  (-3.20 to -0.98; p=0.0002)  - Birthweight (kg)->Systolic blood pressure (53): B=-2.57 mm Hg  (-4.00 to -1.14; p=0.0005)  - Birthweight (kg)->Diastolic blood pressure (36): B=-0.28 mm Hg  (-1.13 to 0.58; p=0.5)  - Birthweight (kg)->Diastolic blood pressure (43): B=-0.48 mm Hg  (-1.35 to 0.39; p=0.5)  - Birthweight (kg)->Diastolic blood pressure (53): B=-0.48 mm Hg  (-1.35 to 0.39; p=0.5)  - Childhood manual social class (vs non-manual) (4)->Systolic blood pressure (36): B=2.09 mm Hg (0.99 to 3.19; p=0.0002)  - Childhood manual social class (vs non-manual) (4)->Systolic blood pressure (43): B=2.50 mm Hg (1.33 to 3.68; p<0.0001)  - Childhood manual social class (vs non-manual) (4)->Systolic blood pressure (53): B=3.91 mm Hg (2.40 to 5.43; p<0.0001)  - Childhood manual social class (vs non-manual) (4)->Diastolic blood pressure (36): B=1.10 mm Hg (0.20 to 2.00; p=0.02)  - Childhood manual social class (vs non-manual) (4)->Diastolic blood pressure (43): B=1.69 mm Hg (-0.79 to 2.59; p=0.0002)  - Childhood manual social class (vs non-manual) (4)->Diastolic blood pressure (53): B=1.93 mm Hg (-1.02 to 2.85; p<0.0001)  - Birthweight (kg)->Systolic blood pressure (36-53): B=-0.4 mm Hg (-1.3 to 0.5; p=0.3) per 10-year increase in age  - Childhood manual social class (vs non-manual) (4)->Systolic blood pressure (36-53): B=1.0 mm Hg (0.01 to 0.19; p=0.03) per 10-year increase in age | Age; gender; birthweight; childhood social class. |

| Supplemental table 5. (cont.) Key characteristics of main relevant studies. | | | |
| --- | --- | --- | --- |
| **Reference** | **Country; Study (period)** | **Exposure (age)->Outcome (age): strength of the association (SE/CI95%)** | **Adjusted confounding (age)** |
| Hardy et al. (2006)^20^ | Finland UK, Faroe Islands; European Birth-Lifecourse-Studies (born 1927-1966) | Men:  - Lower birthweight->Higher systolic blood pressure (31-62): B=0.4 (-4.8 to 5.5) to B=-2.1 (-3.8, -0.4)  Women:  - Lower birthweight->Higher systolic blood pressure (31-62): B=-1.6 (-4.0 to 0.9) to B=-2.1 (-3.0 to -0.1) | Mother’s age, height and education; birth order; current BMI and height. |
| Henderson et al. (2009)^21^ | Scotland; The Aberdeen Children of the 1950s | - Often appears miserable or unhappy (6-12)->Permanently sick or disabled (46-51): OR=3.81 (1.01 to 14.4) | Year of birth, gender, IQ and father’s social class (6-12). |
| Henderson et al. (2012)^22^ | Great Britain; NSHD (born 1946)/NCDS (born 1958)/BCS70 (born 1970) | - NSHD: Higher cognitive ability (10/11)->Long-term sickness (53): OR=0.70 (0.56 to 0.86)  - NCDS: Higher cognitive ability (10/11)->Long-term sickness (42): OR=0.69 (0.61 to 0.77)  - BCS70: Higher cognitive ability (10/11)->Long-term sickness (34): OR=0.80 (0.66 to 0.97) | Gender and parental social class. |

| Supplemental table 5. (cont.) Key characteristics of main relevant studies. | | | |
| --- | --- | --- | --- |
| **Reference** | **Country; Study (period)** | **Exposure (age)->Outcome (age): strength of the association (SE/CI95%)** | **Adjusted confounding (age)** |
| Humphreys et al. (2018)^23^ | England; Hertfordshire Cohort Study/ Clinical Outcomes Study | - Lower birthweight->Multimorbidity (64–68): unadjusted OR=1.29 (0.58, 2.89)  - Higher no. of childhood illnesses->Multimorbidity (64–68): adjusted OR=1.15 (1.06, 1.25) | Diphtheria immunised; no. of childhood illnesses; paternal social class; maternal age at birth; breastfeeding; birthweight; growth in the 1^st^ year; age; gender; adult BMI; adult physical activity; adult smoking and alcohol consumption. |
| Johnston et al. (2019)^24^ | Aberdeen Children of the 1950s (ACONF) cohort (Scotland) | - SES associated with multimorbidity both in adjusted and unadjusted models (p<0.001), however after adjustment association between individual categories has attenuated (e.g.,):  Unskilled vs skilled manual social class at birth->Multimorbidity (mean age: 48): unadjusted OR=1.43 (1.06, 1.93); adjusted OR=1.20 (0.91, 1.70) | Educational attainment, gender, cognition at age 7 and school type. |

| Supplemental table 5. (cont.) Key characteristics of main relevant studies. | | | |
| --- | --- | --- | --- |
| **Reference** | **Country; Study (period)** | **Exposure (age)->Outcome (age): strength of the association (SE/CI95%)** | **Adjusted confounding (age)** |
| Lebenbaum et al. (2018)^25^ | Canada; National Population Health Survey (1996-7); Canadian Community Health Surveys (2012–13) | - Class II/III obesity (mean=45-47)  ->Multimorbidity (mean=45-47): OR= 3.91 (3.06, 4.99)  - Class I obesity (mean=45-47)  ->Multimorbidity (mean=45-47): OR=2.30 (1.94, 2.74)  Changes over time (1996–7 vs 2012-3):  - Class II/III obesity (mean=45-47)  ->Multimorbidity (mean=45-47): OR=1.48 (1.13, 1.95)  - Class I obesity (mean=45-47)  ->Multimorbidity (mean=45-47): OR=1.38 (1.14, 1.68) | Age, gender, marital status, immigrant status, home  ownership, rural residence, education, income quintile, smoking status, alcohol consumption. |
| Li et al. (2015)^26^ | Great Britain; NSHD (born 1946)/NCDS (born 1958) | Men:  Associated only in NCDS:  - NCDS: BMI (7-16)->SBP (43-45): r=0.21 (0.17 to 0.24)  - NSHD: BMI (7-16)->SBP (43-45): r=0.04 (-0.03 to 0.12)  Women:  Stronger association in NCDS than NSHD:  - NCDS: BMI (7-16)->SBP (43-45): r=0.19 (0.12 to 0.26)  - NSHD: BMI (7-16)->SBP (43-45): r=0.11 (0.02 to 0.21) | Blood pressure device; medication; age at examination. |

| Supplemental table 5. (cont.) Key characteristics of main relevant studies. | | | |
| --- | --- | --- | --- |
| **Reference** | **Country; Study (period)** | **Exposure (age)->Outcome (age): strength of the association (SE/CI95%)** | **Adjusted confounding (age)** |
| Mensah & Hobcraft (2007)^27^ | Great Britain; NCDS (born 1958)/BCS70 (born 1970) | - Low birthweight (<=2.5kg)->Fair/poor general health (30/33): 1.16 (0.99 to 1.36)  - Low birthweight (<=2.5kg)->Long-standing illness (30/33): 1.18 (0.96 to 1.46)  - More behaviour problems (5-16)->Fair/poor general health (30/33): 1.40 (1.18 to 1.66)  - More behaviour problems (5-16)->Long-standing illness (30/33): 1.29 (1.03 to 1.62)  - Lower academic test scores (5-16)->Fair/poor general health (30/33): 1.48 (1.25 to 1.75) - 1.89 (1.64 to 2.18)  - Lower academic test scores (5-16)->Long-standing illness (30/33): 1.30 (1.12 to 1.52) | Socioeconomic deprivation, family housing tenure, family disruption, Parental interest, Academic test scores, cohort gender; other predictors as appropriate. |
| Neeleman et al. (2002)^28^ | Great Britain; NSHD (born 1946) | - Negative affect (13)->Somatic symptom count (43): B=0.090 (0.036, 0.144)  - Negative affect (13)->Psychiatric symptom score (43): B=0.130 (0.090, 0.170)  - Anxiety (15)->Somatic symptom count (43): B (boys)=0.110 (0.051, 0.169);  B (girls)=0.044 (-0.018, 0.106)  - Anxiety (15)->Psychiatric symptom score (43): B=0.066 (0.023, 0.109)  - Aggression (13)->Somatic symptom count (43): B=0.052 (0.013, 0.093)  - Aggression (13)->Psychiatric symptom count (43): B=0.046 (0.006, 0.086) | Gender. |
| Park et al. (2012)^29^ | Systematic review (n=39) | - BMI/obesity (2-19)->Type 2 diabetes: OR ranged 1.22-2.04  - BMI/obesity (2-19)->Hypertension: OR ranged 1.35-3.75  - BMI/obesity (2-19)->Coronary heart disease: OR ranged 1.53-5.43 | A range of confounding adjusted in individual studies. |
| Singh-Manoux et al. (2005)^30^ | England; Whitehall II (born 1930-1950) | - Low childhood socioeconomic position (<16)->Coronary heart disease (47-69): RR=1.95 (1.36, 2.81)  - Low childhood socioeconomic position (<16)->Physical component score (47-69): RR=1.30 (0.98, 1.74)  - Low childhood socioeconomic position (<16)->Mental component score (47-69): RR=1.69 (1.26, 2.26)  - Low childhood socioeconomic position (<16)->General Health Questionnaire (47-69): RR=1.52 (1.14, 2.03)  - Low childhood socioeconomic position (<16)->Self-rated health (47-69): RR=1.69 (1.17, 2.44) | Age; cognitive ability (47-69). |

Supplemental text 1. Missing data strategy.

Multimorbidity outcome was missing in 3,793 individuals (47.7% of the used sample), this includes those who had any individual condition missing. Missing data were replaced using multiple imputation. Multiple imputation works under the missing at random (MAR) assumption, which implies that systematic differences between the missing and observed values can be explained by the observed data.^31 32^ The estimates can be unbiased even with up to 90% missing data, provided that the imputation model is correctly specified and the data are Missing-at-Random.^33^ All variables used in the analysis were included in the imputation model. Some of these variables (e.g., BMI at age 10, mental health at age 16) were predictive of missingness (see supplemental table 6 for the estimates) and having them in the imputation model increased the plausibility of the outcome being MAR. The precision of the model was further improved by including variables with very little missing data (less than <1%) collected at birth (e.g., smoking during pregnancy, birth marital status, gender). Finally, we enriched the imputation model and further maximised the plausibility of the MAR assumption with auxiliary variables (self-perceived general health, individual health conditions under multimorbidity outcome and smoking), which were not part of the substantive model of interest, but they were related to the probability of missingness and/or related to the incomplete outcome itself (see supplemental table 6).

| Supplemental table 6. Frequency and predictors of missing data in the exposures and the outcome. | | | |
| --- | --- | --- | --- |
|  | Prevalence of missing data | Predictors of missing data in multimorbidity | Predictors of multimorbidity |
| N=7951 | n (%) | RR (95%CI) | RR (95%CI) |
| Chronic fatigue syndrome (46-48) | 1 (<0.01) | - | - |
| Arthritis (46-48) | 1 (<0.01) | - | - |
| Stroke (46-48) | 1 (<0.01) | - | - |
| Heart problems (46-48) | 1 (<0.01) | - | - |
| Eyes problems (46-48) | 122 (1.5) | - | - |
| Hearing problems (46-48) | 1 (<0.01) | - | - |
| Recurrent back problems (46-48) | 1 (<0.01) | - | - |
| AUDIT-PC (46-48) | 992 (12.5) | - | - |
| Hypertension (46-48) | 1675 (21.1) | - | - |
| Diabetes (46-48) | 2389 (30.1) | - | - |
| Malaise Inventory (46-48) | 738 (9.2) | - | - |
| Asthma/bronchitis (46-48) | 1 (<0.01) | - | - |
| Convulsion, fit, epileptic seizure (46-48) | 1 (<0.01) | - | - |
| Cancer or leukaemia (46-48) | 1 (<0.01) | - | - |
| Multimorbidity at age 46-48 | 3793 (47.7) | 1.00 | 1.00 |
| Being a man | 0 (0) | 1.00 (0.95, 1.05) | 1.25 (1.14, 1.37) |
| Breastfed (never breastfed – reference) | 1,172 (14.7) | 0.93 (0.88, 0.98) | 0.93 (0.84, 1.03) |
| Mother smoked during pregnancy (never smoked – reference) | 44 (0.6) | 1.06 (1.01, 1.11) | 1.25 (1.14, 1.37) |
| Mother’s birth marital status (non-married – reference) | 8 (0.1) | 0.92 (0.83, 1.01) | 0.81 (0.67, 0.97) |
| Mother’s height | 64 (0.8) | 0.99 (0.99, 1.00) | 0.99 (0.99, 1.00) |
| Mother’s age at birth | 46 (0.6) | 1.00 (0.99, 1.00) | 0.99 (0.98, 1.00) |
| Birthweight | 2 (0.03) | 0.88 (0.84, 0.92) | 0.91 (0.83, 1.00) |
| Father’s manual occupational class at birth (non-manual – reference) | 546 (6.9) | 1.16 (1.09, 1.23) | 1.09 (0.97, 1.24) |
| BMI at age 10/11 | 1935 (24.3) | 1.03 (1.01, 1.04) | 1.03 (1.01, 1.06) |
| Cognitive ability problems at age 10/11 | 1751 (22.0) | 0.86 (0.84, 0.88) | 0.95 (0.90, 1.01) |
| Internalising problems at age 16 | 3152 (39.6) | 1.03 (1.00, 1.05) | 1.04 (0.99, 1.09) |
| Externalising problems at age 16 | 3152 (39.6) | 1.05 (1.03, 1.07) | 1.08 (1.04, 1.12) |
| Poor self-perceived general health at age 46-48 | 5 (0.1) | 1.40 (1.33, 1.47) | 2.55 (2.34, 2.77) |
| Ever smoked cigarettes regularly (ever – reference) at age 46-48 | 0 (0) | 0.96 (0.91, 1.02) | 1.28 (1.17, 1.41) |
|  | | | |

| Supplemental table 7. Multimorbidity prevalence at age 46-48 based on different sample definitions. | |
| --- | --- |
| Sample definition | % prevalence |
| Participated at age 46-48 (n total=7951; imputed n=3793‬)^1^ | 33.8 (32.6, 35.0) |
| Alive and not permanent emigrants at age 46-48 (n total=15821; imputed n=11575)^1^ | 35.9 (34.9, 37.0) |
| Complete cases only (n total=4158) | 30.5 (29.1, 31.9) |
| Participated at age 46-48 and had no missing values on objectively measured health outcomes (n total=4963; imputed n=1170)^2^ | 32.8 (31.4, 34.2) |
| Participated at age 46-48 and had no missing values on self-reported health outcomes (n total=6368; imputed n=2122)^2^ | 32.3 (31.1, 33.5) |
| ^1^50 imputations used.  ^2^20 imputations used. | |

A somewhat higher estimate of multimorbidity prevalence was produced (35.9%, 34.9 to 37.0) based on the sample including all those who were alive and were not permanent emigrants from Britain by age 46-48, irrespectively if they participated in the data sweep at this age (n=15821). This sample was the most generalisable to the population of mid-life individuals. However, it was also most severely affected by attrition and non-response, which led to higher estimates as those with missing information were more likely to be of poor health (see supplemental table 6).

| Supplemental table 8. The risk ratio and E-values for the association between each exposure and multimorbidity at age 46-48 in the most adjusted models. | | |
| --- | --- | --- |
|  | Risk Ratio (95%CI) | E-value  (point estimate) |
| Father’s social class at birth (unskilled vs professional) | 1.43 (1.18, 1.74) | 2.21 |
| Birthweight | 0.90 (0.84, 0.96) | 1.46 |
| Cognitive ability (age 10) | 0.96 (0.91, 1.00) | 1.25 |
| BMI (age 10) | 1.03 (1.01, 1.05) | 1.21 |
| Internalising problems (age 16) | 1.04 (1.00, 1.08) | 1.24 |
| Externalising problems (age 16) | 1.06 (1.03, 1.09) | 1.31 |

| Supplemental table 9. Association between early-life exposures and multimorbidity at age 46-48 in the sample with complete cases only and using a multimorbidity definition without mental health morbidity. | | | |
| --- | --- | --- | --- |
|  | Relative risk (95%CI) | | |
|  | Complete case analysis – gender-adjusted (n sample size) | Complete case analysis – confounding-adjusted (n sample size) | Multimorbidity outcome excluding mental health morbidity  (imputed sample n=7951) |
| Father’s SES at birth (age 0)^1^ | (n=3896) | NA |  |
| I – professional | 1.00 | - | 1.00 |
| II – managerial and technical | 1.14 (0.90, 1.45) | - | 1.13 (1.01, 1.25) |
| III – skilled non-manual/manual | 1.24 (1.00, 1.53) | - | 1.19 (1.08, 1.31) |
| IV – partly-skilled | 1.31 (1.03, 1.67) | - | 1.29 (1.16, 1.43) |
| V – unskilled | 1.31 (0.98, 1.75) | - | 1.30 (1.16, 1.46) |
| Birthweight (age 0)^2^ | 0.89 (0.82, 0.98)  (n=4157) | 0.95 (0.86, 1.05)  (n=3835) | 0.90 (0.87, 0.94) |
| Cognitive ability (age 10)^3^ | 0.94 (0.89, 1.00)  (n=3262) | 1.01 (0.92, 1.10)  (n=1763) | 0.95 (0.93, 0.97) |
| BMI (age 10)^4^ | 1.03 (1.01, 1.06)  (n=3226) | 1.03 (1.00, 1.07)  (n=1763) | 1.02 (1.01, 1.03) |
| Internalising problems (age 16)^5^ | 1.06 (1.01, 1.11)  (n=2647) | - 1. (0.93, 1.10)   (n=1232) | 1.00 (0.98, 1.02) |
| Externalising problems (age 16)^6^ | 1.09 (1.04, 1.13)  (n=2640) | 1.04 (0.97, 1.11)  (n=1232) | 1.02 (1.01, 1.04) |
| ^1^ Adjusted for gender.  ^2^ Adjusted for gender, father’s social class at birth, mother ever smoked during pregnancy, mother’s height, mother’s marital status at birth, and mother being a teen at birth.  ^3^ Adjusted for gender, father’s social class at birth, birthweight, mother ever smoked during pregnancy, mother breastfed, mother’s height, mother’s marital status at birth, mother being a teen at birth, household tenure (age 5-10), parental interest in child’s education (age 10), overcrowding (age 5), length of time absent from school due to illness (age 10), parental divorce (age 0-16), mother’s mental health (age 10), and BMI (age 10).  ^4^ Adjusted for gender, father’s social class at birth, birthweight, mother ever smoked during pregnancy, mother breastfed, mother’s height, mother’s marital status at birth, mother being a teen at birth, household tenure (age 5-10), parental interest in child’s education (age 10), overcrowding (age 5), length of time absent from school due to illness (age 10), parental divorce (age 0-16), mother’s mental health (age 10), and cognitive ability (age 10).  ^5^ Adjusted for gender, father’s social class at birth, birthweight, mother ever smoked during pregnancy, mother breastfed, mother’s height, mother’s marital status at birth, mother being a teen at birth, household tenure (age 5-10), parental interest in child’s education (age 10), overcrowding (age 5), length of time absent from school due to illness (age 10), parental divorce (age 0-16), mother’s mental health (age 10), cognitive ability (age 10), BMI (age 10), and externalising problems (age 16).  ^6^ Adjusted for gender, father’s social class at birth, birthweight, mother ever smoked during pregnancy, mother breastfed, mother’s height, mother’s marital status at birth, mother being a teen at birth, household tenure (age 5-10), parental interest in child’s education (age 10), overcrowding (age 5), length of time absent from school due to illness (age 10), parental divorce (age 0-16), mother’s mental health (age 10) cognitive ability (age 10), BMI (age 10), and internalising problems (age 16). | | | |

**References**

1. Babor TF, Higgins-Biddle JC, Saunders JB, et al. AUDIT: The Alcohol Use Disorders Identification Test. Guidelines for Use in Primary Care. Geneva, Switzerland: World Health Organization, 2001.

2. Gómez A, Conde A, Santana JM, et al. Diagnostic usefulness of brief versions of Alcohol Use Disorders Identification Test (AUDIT) for detecting hazardous drinkers in primary care settings. *Journal of Studies on Alcohol* 2005;66:305-08.

3. Rutter M, Tizard J, Whitmore K. Education, Health and Behaviour. London: Longmans 1970.

4. Rodgers B, Pickles A, Power C, et al. Validity of the Malaise Inventory in general population samples. *Soc Psychiatry Psychiatr Epidemiol* 1999;34(6):333-41. [published Online First: 1999/07/28]

5. Elliott J, Johnson J, Shepherd P. National Child Development Study User Guide to the Biomedical Survey 2002-2004 Dataset. London: Centre for Longitudinal Studies, Institute of Education, 2008.

6. American Diabetes Association. Executive summary: standards of medical car in diabetes – 2010. *Diabetes Care* 2010;33:S4-S10.

7. Gillett MJ. International Expert Committee report on the role of the A1c assay in the diagnosis of diabetes: Diabetes Care 2009; 32(7): 1327-1334. *Clin Biochem Rev* 2009;30(4):197-200. [published Online First: 2009/12/17]

8. Elliott C, Murray D, Pearson L. British Ability Scales. . Windsor: National Foundation for Educational Research, 1978.

9. Schoon I. Childhood Cognitive Ability and Adult Academic Attainment: Evidence from Three British Cohort Studies. *Longitudinal and Life Course Studies* 2010;1(3):241-58.

10. Cohort and Longitudinal Studies Enhancement Resources. Harmonised Height, Weight and BMI in Five Longitudinal Cohort Studies: Avon Longitudinal Study of Parents and Children: Special Licence Access.2017.

11. Ploubidis GB, Sullivan A, Brown M, et al. Psychological distress in mid-life: evidence from the 1958 and 1970 British birth cohorts. *Psychol Med* 2017;47(2):291-303. doi: 10.1017/S0033291716002464 [published Online First: 2016/10/14]

12. Wood N, Stafford M, O’Neill D. CLOSER work package 9: Harmonised childhood environment and adult wellbeing measures user guide. London: CLOSER, 2019.

13. Belbasis L, Savvidou MD, Kanu C, et al. Birth weight in relation to health and disease in later life: an umbrella review of systematic reviews and meta-analyses. *Bmc Med* 2016;14 doi: 10.1186/s12916-016-0692-5

14. Birnie K, Cooper R, Martin RM, et al. Childhood Socioeconomic Position and Objectively Measured Physical Capability Levels in Adulthood: A Systematic Review and Meta-Analysis. *Plos One* 2011;6(1) doi: 10.1371/journal.pone.0015564

15. Booth HP, Prevost AT, Gulliford MC. Impact of body mass index on prevalence of multimorbidity in primary care: cohort study. *Fam Pract* 2014;31(1):38-43. doi: 10.1093/fampra/cmt061

16. Buchanan A, Flouri E, Ten Brinke J. Emotional and behavioural problems in childhood and distress in adult life: risk and protective factors. *Aust N Z J Psychiatry* 2002;36(4):521-7. doi: 10.1046/j.1440-1614.2002.01048.x [published Online First: 2002/08/10]

17. Cooper R, Power C. Sex differences in the associations between birthweight and lipid levels in middle-age: Findings from the 1958 British birth cohort. *Atherosclerosis* 2008;200(1):141-49. doi: 10.1016/j.atherosclerosis.2007.11.011

18. Galobardes B, Smith GD, Lynch JW. Systematic review of the influence of childhood socioeconomic circumstances on risk for cardiovascular disease in adulthood. *Ann Epidemiol* 2006;16(2):91-104. doi: 10.1016/j.annepidem.2005.06.053

19. Hardy R, Kuh D, Langenberg C, et al. Birthweight, childhood social class, and change in adult blood pressure in the 1946 British birth cohort. *Lancet* 2003;362(9391):1178-83. doi: Doi 10.1016/S0140-6736(03)14539-4

20. Hardy R, Sovio U, King VJ, et al. Birthweight and blood pressure in five European birth cohort studies: an investigation of confounding factors. *Eur J Public Health* 2006;16(1):21-30. doi: 10.1093/eurpub/cki171

21. Henderson M, Hotopf M, Leon DA. Childhood temperament and long-term sickness absence in adult life. *Brit J Psychiat* 2009;194(3):220-23. doi: 10.1192/bjp.bp.107.044271

22. Henderson M, Richards M, Stansfeld S, et al. The association between childhood cognitive ability and adult long-term sickness absence in three British birth cohorts: :a cohort study. *Bmj Open* 2012;2(2) doi: 10.1136/bmjopen-2011-000777

23. Humphreys J, Jameson K, Cooper C, et al. Early-life predictors of future multi-morbidity: results from the Hertfordshire Cohort. *Age Ageing* 2018;47(3):474-78. doi: 10.1093/ageing/afy005

24. Johnston MC, Black C, Mercer SW, et al. Impact of educational attainment on the association between social class at birth and multimorbidity in middle age in the Aberdeen Children of the 1950s cohort study. *Bmj Open* 2019;9(1) doi: 10.1136/bmjopen-2018-024048

25. Lebenbaum M, Zaric GS, Thind A, et al. Trends in obesity and multimorbidity in Canada. *Prev Med* 2018;116:173-79. doi: 10.1016/j.ypmed.2018.08.025 [published Online First: 2018/09/09]

26. Li L, Hardy R, Kuh D, et al. Life-course body mass index trajectories and blood pressure in mid life in two British birth cohorts: stronger associations in the later-born generation. *International Journal of Epidemiology* 2015;44(3):1018-26. doi: 10.1093/ije/dyv106

27. Mensah FK, Hobcraft J. Childhood deprivation, health and development: associations with adult health in the 1958 and 1970 British prospective birth cohort studies. *J Epidemiol Commun H* 2008;62(7):599-606. doi: 10.1136/jech.2007.065706

28. Neeleman J, Sytema S, Wadsworth M. Propensity to psychiatric and somatic ill-health: evidence from a birth cohort. *Psychol Med* 2002;32(5):793-803. [published Online First: 2002/08/13]

29. Park MH, Falconer C, Viner RM, et al. The impact of childhood obesity on morbidity and mortality in adulthood: a systematic review. *Obes Rev* 2012;13(11):985-1000. doi: 10.1111/j.1467-789X.2012.01015.x [published Online First: 2012/06/27]

30. Singh-Manoux A, Ferrie JE, Lynch JW, et al. The role of cognitive ability (intelligence) in explaining the association between socioeconomic position and health: Evidence from the Whitehall II prospective cohort study. *Am J Epidemiol* 2005;161(9):831-39. doi: 10.1093/aje/kwi109

31. Collins LM, Schafer JL, Kam CM. A comparison of inclusive and restrictive strategies in modern missing data procedures. *Psychological Methods* 2001;6(4):330-51. [published Online First: 2002/01/10]

32. Little R, Rubin DB. Statistical analysis with missing data. Hoboken, N.J: Wiley 2002.

33. Madley-Dowd P, Hughesa R, Tilling K, et al. The proportion of missing data should not be used to guide decisions on multiple imputation. *Journal of Clinical Epidemiology* 2019;110:63-73.
